# GAIT SPEED IMPLEMENTATION IN TWO GERIATRIC CLINICS IS CONSISTENT ACROSS PATIENTS AND VISIT TYPES

**DOI:** 10.1093/geroni/igae098.1913

**Published:** 2024-12-31

**Authors:** Emma Baillargeon, Nancy Jones, Andrea Rosso, Jennifer Brach

**Affiliations:** University of Pittsburgh, Pittsburgh, Pennsylvania, United States; University of Pittsburgh, Pittsburgh, Pennsylvania, United States; University of Pittsburgh, Pittsburgh, Pennsylvania, United States; University of Pittsburgh, Pittsburgh, Pennsylvania, United States

## Abstract

We are implementing gait speed assessment in two outpatient geriatric clinics of a large, academic hospital system in an ongoing quality improvement project. We trained rooming staff (Clinic A: n=5; Clinic B: n=4) to measure 4-meter gait speed using a stopwatch and document the measured speed, or reason for not measuring, in the electronic health record (EHR). The project has been active since 11/1/2023 in Clinic A and 1/19/2024 in Clinic B. EHR data are analyzed monthly to track progress; data through 2/29/24 are included here. We tested whether patient (age, sex) and visit (type, ICD-10) factors were associated with gait speed documentation (yes/no) and measured gait speed. Documentation was completed in 23-58% of visits-per-month for a total of 621 visits (542 patients) in Clinic A and 186 visits (173 patients) in Clinic B. Whether gait speed was documented was not associated with patient age (p=0.3), sex (p=0.2), or appointment type (p=0.2). Gait speed was negatively associated with patient age (R=-0.24, p<0.001) but did not differ by patient sex (p=0.2) or appointment type (p=0.5). Gait speed documentation was not associated with an abnormality of gait and mobility diagnosis (ICD-10 R26; p=0.8), but gait speed was slower in visits with this diagnosis (median (IQR): 0.60m/s (0.51,0.75)) than without (0.69m/s (0.56,0.86), p<0.001). Gait speed documentation is feasible and is being used similarly across patients and visit types. Ongoing data will be assessed as available to inform our goal of increasing the reach and adoption of gait speed assessment in geriatric primary care.

